# Nutrients and Risk of Colon Cancer

**DOI:** 10.3390/cancers2010051

**Published:** 2010-02-10

**Authors:** Jinfu Hu, Carlo La Vecchia, Eva Negri, Les Mery

**Affiliations:** 1 Evidence and Risk Assessment Division, Centre for Chronic Disease Prevention and Control, Public Health Agency of Canada, 785 Carling Avenue, AL: 6807B, Ottawa, Ontario K1A 0K9 Canada; E-Mail: Les.mery@phac-aspc.gc.ca (L.M.); 2 Istituto di Ricerche Farmacologiche “Mario Negri,” Via La Masa, 19-20156 Milan, Italy; E-Mail: lavecchia@marionegri.it (L.V.C.); evanegri@marionegri.it (E.N.); 3 Istituto di Statistica Medica e Biometria, Università degli Studi di Milano, Via Venezian, 1, 20133 Milan, Italy

**Keywords:** unconditional logistic regression, odds ratio, protein, fat, cholesterol, carbohydrate

## Abstract

Dietary fats are thought to be important in the etiology of colon cancer. However, the evidence linking them is inconclusive. Studies on dietary protein, cholesterol and carbohydrate and the risk of colon cancer are also inconsistent. This study examined the association between dietary intake of protein, fats, cholesterol and carbohydrates, and the risk of colon cancer. Mailed questionnaires were completed by 1731 individuals with histologically confirmed cases of colon cancer and 3097 population controls between 1994 and 1997 in seven Canadian provinces. Measurements included socio-economic status, lifestyle habits and diet. A 69-item food frequency questionnaire was used to provide data on eating habits from two years before the study. Odds ratios (OR) and 95% confidence intervals (CI) were computed using unconditional logistic regression. The nutrients were categorized by quartiles based on the distributions among the controls. Intake of polyunsaturated fat, trans-fat and cholesterol were significantly associated with the risk of colon cancer; the ORs for the highest quartiles were 1.36 (95% CI, 1.02–1.80), 1.37 (95% CI, 1.10–1.71) and 1.42 (95% CI, 1.10–1.84), respectively. The association was stronger with proximal colon cancer (PCC). An increased risk was also observed with increasing intake of sucrose for both proximal and distal colon cancers; the ORs for the highest quartiles were 1.67 (95% CI, 1.22–2.29) for PCC and 1.58 (95% CI, 1.18–2.10) for distal colon cancer (DCC). An elevated risk of PCC was also found with increased lactose intake. Our findings provide evidence that a diet low in fat and sucrose could reduce the risk of various colon cancers.

## 1. Introduction

Colorectal cancer ranks the third highest in cancer incidence and fourth in cancer mortality in both sexes combined worldwide. In developed countries, it is the leading site of cancer occurrence in non-smokers of both sexes combined [1]. In Canada, colorectal cancer has the third highest cancer incidence in both men and women, and is the second and third leading cause of cancer-related death in men and women, respectively [2]. With reference to risk factors, considerable attention has focused on diet, and, in particular, overweight and obesity, red meat, processed meat, selected types of fat and alcohol drinking [3,4,5,6]. These factors have been related to increased risk, whereas physical activities have been suggested to reduce the risk of colon cancer. However, the evidence regarding intake of various type of fat and sugar is inconclusive [7].

An ecologic study in Japan showed increased incidence and mortality rates of colon cancer with increased intake of fat and meat [8]. A cross-sectional study from Portugal reported that intake of total fat, saturated fat, trans-fat and cholesterol was positively associated with colorectal cancer [9]. Some case-control studies reported that total fat, monounsaturated fat, polyunsaturated fat and trans-fats were positively associated with colon cancer [10,11], but this was not consistent in other studies [12,13]. Most cohort studies found no significant association between intake of total fat, saturated fat, monounsaturated fatty acids, polyunsaturated fat, trans-fat and the risk of colon and colorectal cancer [14,15,16,17,18,19]. An intervention trial reported that a low-fat dietary pattern did not reduce the risk of colon cancer [20]. The evidence of high consumption of fat increasing the risk of colorectal cancer is therefore inadequate [21]. 

Studies on other nutrients, such as cholesterol, protein and carbohydrate intake and the risk of colon cancer are also open to discussion. A few studies evaluated an association between dietary cholesterol and colon cancer. A few cohort studies found that dietary cholesterol was positively related to colon cancer [17,22], but other studies found no association between cholesterol intake and colorectal cancer [15,16]. Available information on protein and colon cancer is scanty. A case-control study reported that protein intake was positively associated with colon cancer in men [10], whereas other studies found no association [17,19,23]. With reference to carbohydrate intake, some studies reported positive [24] or negative [25] association with colorectal cancer or colon cancer, but most studies found no association between dietary carbohydrate and colon cancer [23,26,27,28]. 

To address these research gaps and inconsistencies, we therefore assessed the role of dietary intake of protein, fats, cholesterol and carbohydrates in colon cancer risk using Canadian data from a nationwide, population-based case-control study, the National Enhanced Cancer Surveillance System (NECSS) [29]. 

## 2. Material and Methods

The NECSS collected individual data from a population-based sample that covered 19 types of cancers and population controls in the Canadian provinces of British Columbia (BC), Alberta (AB), Saskatchewan (SASK), Manitoba (BB), Ontario (ON), Prince Edward Island (PEI), Nova Scotia (NS) and Newfoundland (NFD). The present study did not include cases and controls from Ontario. According to a request from Ontario, the provincial data on colon, rectum and bladder cancers were used by the province only. This decision was made before the project started. 

### 2.1. Cases

Participating provincial cancer registries ascertained a total of 2875 histologically confirmed colon cancer cases of individuals aged 20–76 years between 1994 and 1997. Of these, 427 cases were excluded from the study because 227 patients died and 200 patients were too ill by the time of physician contact. Of 2448 questionnaires sent, 1751 were completed, the response rate being 71.5% of patients contacted. The cases with unclear topography code (n = 20) were excluded. Therefore, this study involved 1731 (964 male and 767 female) histologically confirmed cases of colon cancer as defined by the second edition of the International Classification of Diseases for Oncology (ICDO-2) [30]. Cases of proximal colon cancer (PCC n = 737) included cancer of the caecum, the ascending colon, the hepatic flexure of the colon and the transverse colon; distal colon cancer (DCC n = 994) cases included cancer of the splenic flexure of the colon, the descending colon and the sigmoid colon. 

### 2.2. Controls

Frequency matching was used to select population controls to achieve overall case group with similar age and sex distributions with cases. Provincial cancer registries collected information from controls using the same protocol as for the cases at the same time. The strategies for selecting population controls varied by province, depending on data availability and accessibility. In Prince Edward Island, Nova Scotia, Manitoba, Saskatchewan and British Columbia, age group- and sex-stratified random samples of population were obtained through the provincial health insurance plans. Newfoundland and Alberta used random digit dialling to obtain population samples.

Of 5119 questionnaires sent to potential controls, 81 were returned because of a wrong address; of the remainder, 3097 (1635 men and 1462 women) were completed, yielding a response rate of 61.5% of controls contacted. 

### 2.3. Data collection

The cancer registries were able to identify most cases within one to three months of diagnosis based on pathology reports. After obtaining physician consent, questionnaires were mailed to cases and controls by the cancer registries. If the questionnaire was not completed and returned, a reminder postcard was sent out after 14 days and a second copy of the questionnaire at four weeks. After six weeks, if required, telephone follow-up was used for clarification and completeness. Information was collected on diet, height, weight, smoking history, alcohol drinking, physical activity and socio-economic status.

Data on intake of fats, protein, cholesterol, carbohydrates and energy intake were derived from a food frequency questionnaire (FFQ), which was based on two validated instruments: the short Block questionnaire [31] and the Willett questionnaire [32], with minor modifications to account for differences between the Canadian and the American diets. The FFQ was used to ascertain usual dietary intake two years before the study. The FFQ included 69 specific foods and beverages, and was grouped into eight sections: (a) breads and cereals; (b) meat, poultry, fish, eggs and cheese; (c) vegetables; (d) fruit; (e) sweets; (f) miscellaneous; (g) beverages made with water and (h) other beverages. For each food item, cases and controls were asked to describe how often (per day, per week, per month), on average, they ate the specified serving size. A nutrient database based on the 2005 version of the Canadian Nutrient File was used to estimate nutrient intake and total energy intake [33]. In addition, information on alcohol consumption (beer, wine and spirits) was also collected. 

With reference to the covariates considered in the present study, we collected information on weight “about two years ago”. Body mass index (BMI) was computed as weight (kg) divided by height (m) squared [33]. We defined ever-smokers as subjects who smoked at least 100 cigarettes in their entire life, and current smokers as those who were still smoking one year before the interview. Physical activity two years before the study was based on session frequency, seasons of participation and average time per session for each of 12 categories of the most common types of moderate exercise (including walking, gardening or yard work, home exercise or exercise class, golf, bowling or curling and dancing) and strenuous leisure-time physical activity (including jogging, swimming or water exercise, skiing, cycling or other strenuous exercise).

### 2.4. Statistical Analysis

Unconditional logistic regression was used to estimate odds ratios (OR) and the corresponding 95% confidence intervals (CI). According to our previous studies using the same dataset [35,36], the following potential confounding variables were selected: sex, 10-year age group (<49, 50–59, 60–69, 70–76), province, education (≤8, 9–13, ≥14 years), BMI (<25, 25–29.9, ≥30), alcohol drinking (never drank, ≤1.83, 1.84–10.69, ≥10.70 g/day), moderate activity ( ≤4.63, 4.64–11.83, 11.84–23.96, ≥23.97 hours/month), strenuous activity (≤0.19, 0.20–3.68, ≥3.69 hours/month) total meat (≤4.88, 4.89–7.94, 7.95–12.47, ≥12.48 servings/wk), red meat (≤2.0, 2.1–3.94, 3.95–6.0, ≥6.1 servings/wk), processed meat (≤1.41, 1.42–3.41, 3.42–6.94, ≥6.95 servings/wk), poultry (≤1, 1.1–3, ≥3.1 servings/wk), total consumption of fruit and vegetables (≤14.7, 14.8–22.88, 22.89–31.97, ≥31.97 servings/wk) and total energy intake (≤10644.4, 1044.5–13396.4, 13396.5–16913.13, ≥16913.14 kCal/wk). P value from Chi square was used to measure the distribution between cases and controls for each variable. Tests for trend were assessed for each study variable by substituting the variable in the model in continuous form, and comparing the models without and with the variable of outcome to a chi square distribution with one degree of freedom. Each nutrient intake amount was categorized by quartiles, based on the distribution among controls (Appendix 1). All analyses were made using SAS (Version 9.1) software [37].

## 3. Results and Discussion

### 3.1. Results

Table 1 shows the distribution of 1731 cases of colon cancer and 3097 population controls, and the chi square values according to selected covariates. Cases were older than controls. The proportion of cases reporting overweight or obesity, alcohol drinking (≥10.70 g/d) and pack-years smoking (>30) was significantly higher in the cases than in the controls. In contrast, education (≥14 years), moderate activity (≥23.97 h/month) and strenuous activity (≥0.20 h/month) was significantly higher in the controls than in cases. There was no difference between the cases and the controls regarding family income.

**Table 1:** Description and Chi square of selected covariates for 1731 colon cancer cases and 3097 population-based controls, NECSS, Canada, 1994–1997.

	Cases		Controls		p-value for Chisq
	No.	%	No.	%	
Age					
20–49	191	11.0	838	27.1	<0.0001
50–59	317	18.3	605	19.5	
60–69	747	43.2	1043	33.7	
70–76	476	27.5	611	19.7	
Education (years)					
≤8	319	18.4	471	15.2	<0.0001
9–13	989	57.1	1689	54.5	
≥14	388	22.4	900	29.1	
Family income*					
Low family income	314	18.1	584	18.8	0.51
Lower-middle family income	317	18.3	585	18.9	
Upper-middle family income	414	23.9	779	25.2	
High family income	255	14.7	440	14.2	
Not reported	431	24.9	709	22.9	
Body mass index (kg/m2)					
<25	632	36.5	1461	47.2	<0.0001
25–<30	726	42.0	1176	38.0	
≥30	367	21.2	447	14.4	
Pack-years of smoking					
Never smoked	565	32.6	1123	36.3	<0.0001
≤10	328	19.0	705	22.8	
11–20	289	16.7	470	15.2	
21–30	197	11.4	302	9.8	
>30	325	18.8	447	14.4	
Alcohol drinking (g/d)					
Never drank	632	36.5	1148	37.1	<0.0001
≤1.83	221	12.8	450	14.5	
1.84–10.69	327	18.9	683	22.1	
≥10.70	522	30.2	752	24.3	
Moderate activity (h/month)					
≤4.63	353	20.5	676	21.8	0.04
4.64–11.83	341	19.8	675	21.8	
11.84–23.96	376	21.8	676	21.8	
≥23.97	384	22.3	674	21.8	
Not reported	270	12.7	396	12.8	
Strenuous activity (h/month)					
Never	729	42.3	1146	37.0	0.0001
≤0.19	103	6.0	162	5.2	
0.20–3.68	301	17.5	644	20.8	
≥3.69	299	17.3	647	20.9	
Not reported	292	16.9	498	16.1	

* Household income was indicated as a categorical variable with the following values: Low family income:<$20,000 with ≤ 3 people or $30,000 with ≥4 people; Lower-middle family income: $20,000–$30,000 with ≤3 people or $30,000–<$50,000 with ≥4 people; Upper-middle family income:<$50,000 with ≤3 people or $50,000–$100,000 with ≥4 people; High family income: ≥50,000 for up to 3 people or ≥100,000 for ≥4 people. Total vegetables: tomatoes, carrots, broccoli, cabbage, cauliflower, brussel sprouts, spinach or other greens, yellow squash, green beans, corn, peas or any other vegetable; soups with vegetables, and total fruit: apples, pears, oranges, bananas, cantaloupe or other fruit, fresh or canned. Note: Totals may vary due to missing values.

Table 2 presents the ORs (and the corresponding 95% CIs) for intake of proteins, fats, cholesterol and carbohydrates. Intake of polyunsaturated fat, trans-fats, cholesterol and sucrose was significantly related to the risk of colon cancer; the ORs for the highest versus the lowest quartile were 1.36 (95% CI, 1.02–1.80), 1.37 (95% CI, 1.10–1.71), 1.42 (95% CI, 1.10–1.84) and 1.61 (95% CI, 1.27–2.04), respectively. No significant association between glucose intake and the risk of colon cancer was observed . Likewise, no association was observed with intake of total fat, saturated fat, monounsaturated fat, total proteins, total carbohydrate, lactose, maltose, fructose and galactose, although a significantly increased OR was observed for galactose intake in the second quartile compared with the lowest one.

**Table 2:** Odds ratios* and 95% confidence intervals of fats, protein, cholesterol and carbohydrates for colon cancer, NECSS, Canada, 1994–1997.

Nutrients	Quartiles				P-value
(g/wk)	I (low)	II	III	IV (high)	for trend
Total protein	328/769	449/773	449/775	503/773	
OR (95% CI)	1.0 (ref.)	1.13 (0.91–1.40)	0.92 (0.71–1.21)	0.92 (0.65–1.30)	0.38
Total fat	308/772	440/769	467/775	514/774	
OR (95% CI)	1.0 (ref.)	1.22 (0.99–1.51)	1.18 (0.91–1.54)	1.21 (0.86–1.69)	0.40
Saturated fat	310/769	424/772	466/774	529/775	
OR (95% CI)	1.0 (ref.)	1.21 (0.98–1.50)	1.22 (0.96–1.56)	1.26 (0.93–1.70)	0.20
Monounsaturated fat	314/772	424/770	478/774	513/774	
OR (95% CI)	1.0 (ref.)	1.23 (0.99–1.52)	1.29 (0.99–1.66)	1.31 (0.95–1.82)	0.14
Polyunsaturated fat	333/772	407/771	465/773	524/774	
OR (95% CI)	1.0 (ref.)	1.15 (0.93–1.41)	1.22 (0.97–1.55)	1.36 (1.02–1.80)	0.04
Trans–fat	318/773	397/770	487/773	526/774	
OR (95% CI)	1.0 (ref.)	1.09 (0.89–1.33)	1.29 (1.05–1.59)	1.37 (1.10–1.71)	0.002
Cholesterol (mg/wk)	316/773	401/772	489/772	523/773	
OR (95% CI)	1.0 (ref.)	1.17 (0.95–1.43)	1.31 (1.05–1.63)	1.42 (1.10–1.84)	0.006
Total carbohydrates	347/770	417/772	460/775	505/773	
OR (95% CI)	1.0 (ref.)	1.07 (0.86–1.34)	1.14 (0.84–1.54)	1.15 (0.78–1.71)	0.49
Sucrose	307/771	407/773	454/772	561/774	
OR (95% CI)	1.0 (ref.)	1.18 (0.97–1.44)	1.30 (1.05–1.61)	1.61 (1.27–2.04)	<0.0001
Lactose	384/771	427/772	489/773	729/774	
OR (95% CI)	1.0 (ref.)	1.09 (0.90–1.31)	1.09 (0.90–1.32)	1.01 (0.83–1.24)	0.93
Maltose	334/772	403/773	490/770	502/775	
OR (95% CI)	1.0 (ref.)	1.13 (0.92–1.39)	1.33 (1.07–1.66)	1.24 (0.96–1.59)	0.06
Glucose	386/772	400/774	467/772	476/772	
OR (95% CI)	1.0 (ref.)	1.00 (0.82–1.22)	1.15 (0.93–1.42)	1.22 (0.97–1.54)	0.05
Fructose	387/772	415/772	455/774	472/772	
OR (95% CI)	1.0 (ref.)	1.06 (0.88–1.29)	1.16 (0.94–1.43)	1.25 (0.99–1.57)	0.06
Galactose	314/772	486/770	452/774	477/774	
OR (95% CI)	1.0 (ref.)	1.39 (1.15–1.69)	1.21 (0.99–1.48)	1.20 (0.97–1.50)	0.41

* Adjusted for 10-year age group, sex, province, education, body mass index (<25, 25–29.9, ≥30), moderate activity (hours/month), strenuous activity (hours/month), alcohol drinking (g/d), pack-year smoking, total meat, red meat, processed meat, poultry, total consumption of fruit and vegetables and total energy intake. Total meat: beef, pork, lamb, hamburger, hotdogs, bacon, sausage, smoked meat or corned beef, luncheon meats and liver. Red meat: beef, pork or lam as a main dish, beef and pork or lam as a mixed dish, and hamburger. Processed meat: hotdogs, lunch meat, smoked meat or corned beef, bacon and sausage. Poultry: chicken or turkey. Total consumption of fruit and vegetables is the same as in Table 1 Note: Totals may vary due to missing values.

Table 3 gives the ORs and 95% CIs for intake of proteins, fats, cholesterol and carbohydrates, and the risk of PCC. Intake of polyunsaturated fat, trans-fat, cholesterol, sucrose and lactose was significantly associated with PCC: the ORs for the highest quartile were 1.47 (95% CI = 1.00–2.16), 1.48 (95% CI = 1.09–2.01), 1.50 (95% CI = 1.06–2.13) and 1.67 (95% CI = 1.22–2.29) and 1.36 (95% CI = 1.04–1.79), respectively. An elevated risk of galactose was also seen with the second and fourth quartile, in the absence of significant trend. Intake of total fat, saturated fat, monounsaturated fat, protein, total carbohydrate, maltose, glucose and fructose was not associated with PCC.

**Table 3:** Odds ratios* and 95% confidence intervals of fats, protein, cholesterol and carbohydrates for proximal colon cancer, NECSS, Canada, 1994–1997.

Nutrients	Quartiles				P-value
(g/wk)	I (low)	II	III	IV (high)	for trend
Total protein	138/769	183/773	199/775	217/773	
OR (95% CI)	1.0 (ref.)	1.14 (0.85–1.54)	1.10 (0.76–1.58)	1.16 (0.73–1.86)	0.65
Total fat	123/772	191/769	205/775	218/774	
OR (95% CI)	1.0 (ref.)	1.35 (1.00–1.81)	1.36 (0.95–1.96)	1.37 (0.87–2.18)	0.26
Saturated fat	134/769	161/772	213/774	229/775	
OR (95% CI)	1.0 (ref.)	1.11 (0.83–1.48)	1.37 (0.98–1.90)	1.37 (0.91–2.07)	0.09
Monounsaturated fat	131/772	185/770	205/774	216/774	
OR (95% CI)	1.0 (ref.)	1.25 (0.93–1.67)	1.30 (0.91–1.85)	1.26 (0.81–1.98)	0.37
Polyunsaturated fat	133/772	172/771	207/773	225/774	
OR (95% CI)	1.0 (ref.)	1.20 (0.91–1.60)	1.38 (1.00–1.90)	1.47 (1.00–2.16)	0.05
Trans-fat	122/773	167/770	217/773	231/774	
OR (95% CI)	1.0 (ref.)	1.16 (0.88–1.52)	1.45 (1.09–1.92)	1.48 (1.09–2.01)	0.005
Cholesterol (mg/wk)	133/773	164/772	215/772	225/773	
OR (95% CI)	1.0 (ref.)	1.12 (0.84–1.48)	1.38 (1.02–1.86)	1.50 (1.06–2.13)	0.01
Total carbohydrates	145/770	178/772	207/775	207/773	
OR (95% CI)	1.0 (ref.)	1.05 (0.77–1.42)	1.14 (0.76–1.72)	1.06 (0.62–1.80)	0.82
Sucrose	127/771	172/773	194/772	244/774	
OR (95% CI)	1.0 (ref.)	1.19 (0.90–1.56)	1.32 (0.99–1.77)	1.67 (1.22–2.29)	0.001
Lactose	146/771	173/772	212/773	206/774	
OR (95% CI)	1.0 (ref.)	1.24 (0.95–1.61)	1.29 (0.99–1.68)	1.36 (1.04–1.79)	0.04
Maltose	142/772	175/773	216/770	204/775	
OR (95% CI)	1.0 (ref.)	1.16 (0.88–1.52)	1.31 (0.98–1.76)	1.15 (0.82–1.62)	0.39
Glucose	163/772	176/774	199/772	199/772	
OR (95% CI)	1.0 (ref.)	1.00 (0.76–1.29)	1.09 (0.82–1.45)	1.13 (0.82–1.55)	0.36
Fructose	161/772	185/772	195/774	196/772	
OR (95% CI)	1.0 (ref.)	1.09 (0.84–1.42)	1.13 (0.84–1.50)	1.16 (0.85–1.59)	0.40
Galactose	130/772	192/770	196/774	219/774	
OR (95% CI)	1.0 (ref.)	1.33 (1.02–1.74)	1.24 (0.94–1.64)	1.36 (1.02–1.83)	0.10

* Adjusted for 10-year age group, sex, province, education, body mass index (<25, 25–29.9, ≥30), moderate activity (hours/month), strenuous activity (hours/month), alcohol drinking (g/d), pack-year smoking, total meat, red meat, processed meat, poultry, total consumption of fruit and vegetables and total energy intake. Food groups are the same as in Table 1 and Table 2 Note: Totals may vary due to missing values.

Intake of sucrose was significantly associated with risk of DCC (Table 4); the OR for the highest quartile was 1.58 (95% CI = 1.18–2.10). Significant trends in risk of DCC were observed for trans fat, maltose, glucose and fructose: the ORs for the highest quartile were 1.30 (95% CI, 0.99–1.70), 1.30 (95% CI, 0.96–1.76), 1.31 (95% CI, 0.99–1.73) and 1.31 (95% CI, 0.99–1.73), respectively. No association was observed with intake of total fat, each separate type of fat, except trans-fat, cholesterol, lactose and galactose.

**Table 4:** Odds ratios* and 95% confidence intervals of fats, protein, cholesterol carbohydrates for distal colon cancer, NECSS, Canada, 1994–1997.

Nutrients	Quartiles				P-value
(g/wk)	I (low)	II	III	IV (high)	for trend
Total protein	190/769	266/773	250/775	286/773	
OR (95% CI)	1.0 (ref.)	1.09 (0.84–1.40)	0.79 (0.57–1.10)	0.74 (0.49–1.13)	0.06
Total fat	185/772	249/769	262/775	296/774	
OR (95% CI)	1.0 (ref.)	1.143 (0.87–1.46)	1.06 (0.77–1.47)	1.07 (0.71–1.62)	0.86
Saturated fat	176/769	263/772	253/774	300/775	
OR (95% CI)	1.0 (ref.)	1.29 (1.00–1.67)	1.12 (0.83–1.51)	1.16 (0.80–1.69)	0.72
Monounsaturated fat	183/772	239/770	273/774	297/774	
OR (95% CI)	1.0 (ref.)	1.20 (0.92–1.55)	1.26 (0.92–1.73)	1.32 (0.89–1.96)	0.19
Polyunsaturated fat	200/772	235/771	258/773	299/774	
OR (95% CI)	1.0 (ref.)	1.12 (0.87–1.44)	1.11 (0.83–1.47)	1.26 (0.90–1.78)	0.23
Trans–fat	196/773	230/770	270/773	295/774	
OR (95% CI)	1.0 (ref.)	1.06 (0.83–1.35)	1.21 (0.94–1.55)	1.30 (0.99–1.70)	0.03
Cholesterol (mg/wk)	183/773	237/772	274/772	298/773	
OR (95% CI)	1.0 (ref.)	1.19 (0.93–1.53)	1.24 (0.95–1.63)	1.35 (0.99–1.85)	0.07
Total carbohydrates	202/770	239/772	253/775	298/773	
OR (95% CI)	1.0 (ref.)	1.10 (0.84–1.45)	1.12 (0.77–1.61)	1.23 (0.76–1.97)	0.44
Sucrose	180/771	235/773	260/772	317/774	
OR (95% CI)	1.0 (ref.)	1.19 (0.93–1.52)	1.28 (0.99–1.66)	1.58 (1.18–2.10)	0.002
Lactose	238/771	254/772	277/773	223/774	
OR (95% CI)	1.0 (ref.)	1.01 (0.81–1.27)	0.98 (0.78–1.24)	0.81 (0.63–1.03)	0.09
Maltose	192/772	228/773	274/770	298/775	
OR (95% CI)	1.0 (ref.)	1.10 (0.86–1.41)	1.33 (1.02–1.73)	1.30 (0.96–1.76)	0.05
Glucose	223/772	224/774	268/772	277/772	
OR (95% CI)	1.0 (ref.)	0.97 (0.76–1.23)	1.21 (0.94–1.56)	1.31 (0.99–1.73)	0.02
Fructose	226/772	230/772	260/774	276/772	
OR (95% CI)	1.0 (ref.)	1.03 (0.81–1.30)	1.20 (0.93–1.54)	1.31 (0.99–1.73)	0.03
Galactose	184/772	294/770	256/774	258/774	
OR (95% CI)	1.0 (ref.)	1.45 (1.14–1.83)	1.19 (0.93–1.53)	1.11 (0.85–1.45)	0.93

* Adjusted for 10-year age group, sex, province, education, body mass index (<25, 25–29.9, ≥30), moderate activity (hours/month), strenuous activity (hours/month), alcohol drinking (g/d), pack-year smoking, total meat, red meat, processed meat, poultry, total consumption of fruit and vegetables and total energy intake. Food groups are the same as in Table 1 and Table 2; Note: Totals may vary due to missing values.

### 3.2. Discussion

In this large, nationwide, population-based case-control study, we found that intake of polyunsaturated fat, trans-fat and cholesterol was significantly associated with PCC. An increased risk was also observed with increased intake of sucrose in both sides of colon, lactose in PCC, and glucose and fructose in DCC. No association was found between intake of total fat, saturated fat, monounsaturated fat, protein, maltose, galactose or total carbohydrates and both PCC and DCC.

Most attention thus far has been given to dietary meat and fat intake and the risk of colorectal cancer. A substantial amount of evidence shows that red and processed meat are related to colorectal cancer [7]; the main sources of fat in this study were meat. In the same dataset, consumption of red and processed meat was positively associated with colon cancer [35,36]. In line with these findings, in the present study, dietary polyunsaturated fat and trans-fat were significantly related to the risk of colon cancer. Other case-control studies also reported that intake of total fat, monounsaturated fat and polyunsaturated fat was related to colon cancer [10,11], whereas this was not seen in another study [38]. The association between dietary fat (and types of fat) and the risk of colorectal cancer are inconsistent [39]. Most cohort studies [14,15,16,17,18,19] and a meta-analysis of six prospective studies [40] did not support an association between intake of total fat, saturated fat, monounsaturated fat and polyunsaturated fat and the risk of colon or colorectal cancer. However, a cohort study observed that a high intake of animal fat increased the risk of colon cancer [41]. It remains thus possible that some types of fat could be related to this cancer [42]. 

Studies on trans-fat and colon cancer are sparse. A case-control study showed that trans-fatty acids were related to colorectal cancer [43], but this was not supported by two cohort studies [15,16]. Another study reported that consumption of trans-fatty acids, but not the cis form, was associated with an increased risk of colon cancer, which was stronger in women with PCC [11]. Our results show that an increased intake of trans-fat was related to the risk of both PCC and DCC. Trans-fat appears to impact markers of systemic inflammation [44], and trans–fats adversely affect endothelial function [45]. Patients with inflammatory bowel disease such as ulcerative colitis and Crohn’s disease are at high risk for developing colon cancer [46]. Chronic inflammation and colorectal cancer are closely linked [47]. A cohort study in Korea found that an elevated white blood cell count was associated with an increase in both mortality and incidence rates of colon cancer, and suggested that inflammation increases the risk of colon cancer [48]. An animal model showed the molecular, classical nuclear factor-kB (NF-κB) activation, links between chronic inflammation and colon tumorigenesis [49].

Inconsistent data are available on cholesterol and colon cancer. A small case-control study suggested an association between low blood cholesterol and colorectal cancer [50]. Three cohort studies found that dietary cholesterol was directly related to colon and colorectal cancer [17,22,25]. This finding was consistent with our results, but not with other studies [15,16,51]. The JPHC cohort study indicated that serum cholesterol levels were not related to the risk of colorectal cancer [52]. An animal study reported cholesterol acts as a co-carcinogen in the development of colorectal cancer [53].

A Japanese cohort study observed an increased risk of colon cancer with intake of animal protein in men only [54]. In contrast, an inverse association between protein intake and colorectal cancer was found in women in the US [18] and, specifically, with milk protein in Finnish men [15] and soy protein in Chinese women [55]. No association was shown in other cohort studies [17,19] and in a meta-analysis of animal protein [40], in broad agreement with our results. 

A cohort study reported that dietary carbohydrate intake was inversely associated with the risk of colon cancer in women [25]. In contrast, another cohort study observed that a diet with high dietary carbohydrates was positively related to the risk of colorectal cancer in women [24]. However, several cohort studies [26,27,28,56,57,58,59,60] and meta-analyses [61,62] did not support the hypothesis that carbohydrate intake increases the risk of colon or colorectal cancer. Two cohort studies indicated that total carbohydrate intake was not related to PCC and RCC [27,28], in agreement with our findings. A large Korean cohort study reported that fasting serum glucose level was not related to colorectal cancer in men and women [63], as did a study in Sweden of men and women combined [64]. A case-control study reported that high intake of sucrose increased the risk of colon cancer [65], in agreement with our data. A meta-analysis suggested that subjects who develop colorectal cancer have increased prediagnostic blood levels of insulin and glucose [66]. A plausible mechanism from experimental studies in mice showed that sucrose intake might increase the risk of colon cancer by increasing circulating levels of insulin and insulin-like growth factor-I (IGF-I) [67]. A sucrose-rich diet caused mutations in the rat colon epithelium [68].

Recent evidence indicates that PCC and DCC may have distinct pathogenic mechanisms [69,70]. In the present study, we found that intake of polyunsaturated fat and trans-fat was significantly associated with PCC, but not with DCC. Another study also reported that intake of total fat, monounsaturated fat and saturated fat was associated with PCC, but not with DCC [10]. A cohort study showed that long term high-fat dairy food reduced the risk of DCC, whereas there was no association with PCC [71]. It is possible that some factors play different roles in each side of colon, since proximal and distal segments of the large intestine have different embryologic origins and biologic characteristics, and proximal and distal tumours follow broadly different molecular pathways of carcinogenesis [70,72]. 

Several explanations could be considered from some of the inconsistent results of the relation of diet and colorectal cancer. Retrospective case-control studies on diet and colon cancer could be influenced by recall and selection bias. Some cohort studies involved small number of cases, which could not have adequate power to show significant results. 

Our study is a large, population-based investigation from seven Canadian provinces, based on a widely used and validated FFQ [31]. Nevertheless, possible limitations should be mentioned, in particular, the validity of information on dietary habits two years before the study. The possibility of differential misclassification of exposure cannot be ruled out, but non-differential misclassification between cases and controls would bias the ORs toward unity in most instances [73]. Consequently, the actual risks may be stronger than observed. Cases might report their food intake differently than controls. However, the project was presented to subjects as a “Canadian study of health and the environment”, thus limiting the scope for differential reporting. It has been shown that recall of FFQ data by controls is satisfactorily reproducible [74]. 

Selection bias and confounding are also of concern. About 15% of the cases (who were too ill or had died) were not included in this study. Family history of colon cancer increases risk of colon cancer [75,76], with a stronger association at younger age. In the present study, 11% of cases were under 50 years old. However, we did not collect information on family history of colon cancer. However, we were able to allow in the analyses for a large number of potential confounding factors, including age, province, education, body mass index, alcohol, tobacco, physical activity, meat and energy intake. 

## 4. Conclusions

In the present study, we found that dietary intake of polyunsaturated fat, trans-fat and cholesterol was significantly associated with PCC. An increased risk was also observed with increasing intake of sucrose for both sides of the colon (PCC and DCC), of lactose for PCC, and of glucose and fructose for DCC. No association was found with intake of proteins, maltose, galactose or total carbohydrates in both PCC and DCC. 

The findings of this study provide further evidence that a diet rich in several types of fat and sucrose is associated with a risk of colon cancer. This role may be different in various anatomic sibsites.

## Abbreviations

BMI: body mass index
OR: odds ratio
CI: confidence interval
DCC: distal colon cancer
PCC: proximal colon cancer
FFQ: food frequency questionnaire
IGF-I: insulin-like growth factor-I
JPHC: Japan Public Health-Center cohort study
NECSS: National Enhanced Cancer Surveillance System

## Appendix

**Appendix 1:** Quartile Cut-points for Fats, Protein and Carbohydrates Among Controls, NECSS Study, Canada, 1994–1997.

Nutrients (g/wk)	Quartiles			
	I (low)	II	III	IV (high)
Total proteins	≤356.47	356.48–456.82	456.83–575.25	≥575.26
Total fats	≤313.77	313.78–422.16	422.17–543.23	≥543.24
Saturated fat	≤115.77	115.78–157.81	157.82–205.93	≥205.94
Monounsaturated fat	≤109.48	109.49–148.55	148.56–194.37	≥194.38
Polyunsaturated fat	≤55.59	55.60–74.56	74.57–96.89	≥96.90
Trans–fat	≤6.86	6.87–10.93	10.94–19.07	≥19.08
Cholesterol (mg/wk)	≤966.26	926.27–1412.75	1412.76–1880.27	≥1880.28
Total carbohydrates	1406.49	1406.50–1790.47	1790.48–2253.18	≥2253.19
Sucrose	≤150.76	150.77–214.93	214.94–283.79	≥283.80
Lactose	≤33.02	33.03–93.94	93.95–196.50	≥196.51
Maltose	≤7.56	7.57–10.52	10.53–14.41	≥14.42
Glucose	≤86.46	86.47–123.40	123.41–176.90	≥176.91
Fructose	≤90.88	90.89–133.97	133.98–194.10	≥194.11
Galactose	≤0.47	0.48–0.75	0.76–1.11	≥1.12
